# Evaluating multiple causes of persistent low microwave backscatter from Amazon forests after the 2005 drought

**DOI:** 10.1371/journal.pone.0183308

**Published:** 2017-09-05

**Authors:** Steve Frolking, Stephen Hagen, Bobby Braswell, Tom Milliman, Christina Herrick, Seth Peterson, Dar Roberts, Michael Keller, Michael Palace

**Affiliations:** 1 Institute for the Study of Earth, Oceans, and Space, University of New Hampshire, Durham, NH, United States of America; 2 Department of Earth Sciences, University of New Hampshire, Durham, NH, United States of America; 3 Applied GeoSolutions, Newmarket, NH, United States of America; 4 Department of Geography, University of California, Santa Barbara, CA, United States of America; 5 USDA Forest Service, International Institute of Tropical Forestry, Rio Piedras, Puerto Rico; 6 Jet Propulsion Lab, California Institute of Technology, Pasadena, California, United States of America; 7 Embrapa Agricultural Informatics, Campinas, Brazil; University of Maryland at College Park, UNITED STATES

## Abstract

Amazonia has experienced large-scale regional droughts that affect forest productivity and biomass stocks. Space-borne remote sensing provides basin-wide data on impacts of meteorological anomalies, an important complement to relatively limited ground observations across the Amazon’s vast and remote humid tropical forests. Morning overpass QuikScat Ku-band microwave backscatter from the forest canopy was anomalously low during the 2005 drought, relative to the full instrument record of 1999–2009, and low morning backscatter persisted for 2006–2009, after which the instrument failed. The persistent low backscatter has been suggested to be indicative of increased forest vulnerability to future drought. To better ascribe the cause of the low post-drought backscatter, we analyzed multiyear, gridded remote sensing data sets of precipitation, land surface temperature, forest cover and forest cover loss, and microwave backscatter over the 2005 drought region in the southwestern Amazon Basin (4°-12°S, 66°-76°W) and in adjacent 8°x10° regions to the north and east. We found moderate to weak correlations with the spatial distribution of persistent low backscatter for variables related to three groups of forest impacts: the 2005 drought itself, loss of forest cover, and warmer and drier dry seasons in the post-drought vs. the pre-drought years. However, these variables explained only about one quarter of the variability in depressed backscatter across the southwestern drought region. Our findings indicate that drought impact is a complex phenomenon and that better understanding can only come from more extensive ground data and/or analysis of frequent, spatially-comprehensive, high-resolution data or imagery before and after droughts.

## Introduction

Tropical forests are large reservoirs of carbon and biodiversity, and influence global climate [1–3]. An increased understanding of how drought affects these forests is of prime interest [4–6]. Droughts cause changes in humid tropical forest tree mortality, carbon allocation and gross productivity, and fire frequency [7–10]. The Amazonian rainforest experienced two significant droughts in 2005 and 2010: a severe drought occurred in 2005 in the western Amazon Basin [11] and a more widespread drought occurred in 2010 [12]. Aragão et al. [7] analyzed Tropical Rainfall Monitoring Mission (TRMM) precipitation data for the Amazon Basin for 1998–2005, and report mean annual precipitation of about 2.2m for the period and for 2005, but a mean dry season precipitation deficit in 2005 about 40% greater than the period mean of 140 mm, with dry season rainfall anomalies greater than 1 standard deviation below the mean for much of the southwestern Amazon Basin. Analysis of long-term forest plots documented increased tree mortality over a large area following the 2005 drought with carbon losses estimated between 1.2 and 1.6 Pg C [13]. Carbon losses associated with the 2010 drought were even larger, estimated as high as 2.2 Pg C [12]. Further research has examined the forest response to repeated droughts, looking at net biomass gain and stem mortality [6], and examined how well models simulate observed relationships between aboveground biomass, woody net primary production, and stem mortality [14]. Because of anthropogenic impacts on these forests directly through forest fire, selective logging, forest fragmentation, and land conversion, additional attention to these impacts needs to be used when examining drought in tropical forests [3].

Microwave remote sensing provides a unique view of humid tropical forest systems, with limited contamination from clouds and aerosols. Shorter microwave wavelengths, such as Ku-band, primarily scatter from the upper canopy of dense forests, providing a measure of canopy biometric properties that can be fundamentally different than optical sensors [15–17]. Microwave backscatter from vegetation canopies is sensitive to the geometry or structure of the upper canopy and its dielectric properties, which are dominated by liquid water content [16]. Saatchi et al. [18] estimated a large-footprint canopy Ku-band microwave penetration depth (half-power) of 1–2 m for closed canopy with <5% gap, and 3–6 m for a canopy with 25% gap. Tropical forest canopy Ku-band microwave backscatter is low in the dry season, due to weaker volume scattering from the drier canopy [19]. Frolking et al. [19] showed that Quikscat (QScat) Ku-band scatterometer microwave backscatter from Amazonian tropical forest was anomalously low during the 2005 drought for the early morning overpass data, but not for the late afternoon overpass data. They hypothesized that this was due to widespread reduced nocturnal rehydration of canopy leaves during the drought, and that this signal was not significant during late afternoon overpass because the canopy is typically dry at that time of day in the dry season. Saatchi et al. [18] subsequently showed that, when averaged over an ~80,000 km^2^ forested region where the dry season precipitation anomaly was strongest in 2005 (forested land in a box bounded by 4°S-12°S, 66°W-76°W), the anomalously low morning overpass backscatter persisted through 2009, just before the onset of the 2010 drought. QScat stopped collecting data in Nov. 2009, so these data are not available for the 2010 drought. Normalized backscatter anomalies (Z-scores) for this region were less than -2 for July through October 2005 (drought), and averaged about -1 for 2006–2009, while they ranged from -0.5 to +1 during 2000–2004 (Fig 2b in [18]).

There was negligible QScat backscatter anomaly for the northern Amazon basin during 2000–2009 [18, 19], showing that QScat was a stable instrument through 2000–2009, and that the persistent drop in the southwestern Amazon drought region could not be attributed to instrument drift or bias. Saatchi et al. [18] concluded that this persistent low backscatter may be related to a persistent aspect of slow recovery (>4 y) of forest canopy structure rather than leaf wilting or shedding, from which a more rapid recovery is likely. Maeda et al. [20] confirmed this pattern in the regional QScat backscatter. They also found a very similar pattern in the Total Water Storage anomalies estimated by GRACE (NASA’s Gravity Recovery and Climate Experiment) satellites [21]–i.e., a drought-induced abrupt drop in the anomaly, followed by a slow and steady recovery–for both the 2005 and 2010 droughts, analyzing data over a similar region (5°S-11°S, 70°W-76°W). They also computed a river-basin scale cumulative water deficit anomaly as basin runoff plus change in Total Water Storage. Aggregated for the larger Solimões River basin of the western Amazon, this cumulative water deficit anomaly persisted for two years after both the 2005 and 2010 droughts, despite a general recovery in annual precipitation [20].

Ku-band microwave backscatter also declines with partial loss of humid tropical forest cover [22]. Due to its coarse spatial resolution, QScat is not ideally suited for mapping deforestation at the scale of most individual land use activity in the humid tropics. However, if regional land-cover change activity persists (either forest canopy loss or gain), a signal can emerge from the seasonal and interannual backscatter variability over multiple years [22].

Saatchi et al. [18] concluded that the slow (>4 year) recovery of the regional (>70 Mha) forest canopy from the 2005 drought could have made the forest more susceptible to physiological stress during a subsequent drought in 2010. Using field-plot data from across the Amazon, Feldpausch et al. [6] examined the compounding effect of multiple anomalously dry years and found no evidence that this led to enhanced stand productivity or mortality. In order to better understand the potential vulnerability of this forest region to drought, and the direct impact of drought on the regional forest canopy, we re-analyzed remote sensing data from the southwestern Amazon region, but at a comparatively high degree of spatial detail (0.05° and 0.2° grid cells) rather than in aggregate (8° by 10°), for the years 2001–2009, encompassing the 2005 drought year. We note that the resolution of our study (~10 km), while fine compared to the ~10^3^ km resolution regional backscatter aggregation of Saatchi et al. [18], is still quite large compared to the scale of field plots (generally ~10^−1^ km) or individual trees (~10^−2^ km), and may not fully capture the effects of processes operating at those scales. We considered a set of remote sensing derived measures that could be indicators of drought exposure (precipitation deficit, land surface temperature), or of the resultant direct vegetation sensitivity to this exposure (microwave backscatter, non-photosynthetic vegetation fraction). We also computed several additional metrics of forest canopy and weather change over the decade: QScat backscatter, forest cover loss, and annual dry-season precipitation deficits and temperature anomalies. Since the analyses of Saatchi et al. [18], a new 30-m global forest cover change product has been developed and released [23], which we use in this study both to generate a regional forest mask and to quantify forest cover change. We assess the contribution of several factors that could contribute to the persistent drop in Ku-band morning overpass backscatter reported by Saatchi et al. [18], i.e., drought, land-cover change, and seasonal weather change. It is important to understand how these mechanisms, individually and together, influence canopy vitality. We also compare this region affected by the 2005 drought, and analyzed by Saatchi et al. [18], to adjacent Amazon forest regions to the north and east in order to provide additional context and quality control for our results.

## Materials and methods

Our analysis focused on the 2005 drought impact on a ~800,000 km^2^ forested region in the southwestern Amazon identified as showing persistent low QScat backscatter post-drought [18]. We analyzed a suite of moderate resolution remote sensing datasets that had (1) a period of record which included multiple years pre- and post-drought (i.e., roughly 2001–2009), and (2) which could be expected to provide regional information about the 2005 drought, drought impact on the forest, forest canopy change, and changes in regional seasonal dry season weather patterns between the pre- and post-drought periods. We also expanded the area of analysis to equivalently sized regions to the north and east of the focal study region, to include areas with different magnitudes of drought in 2005 and land use activity during 2001–2009.

### Study area

**Fig 1:**
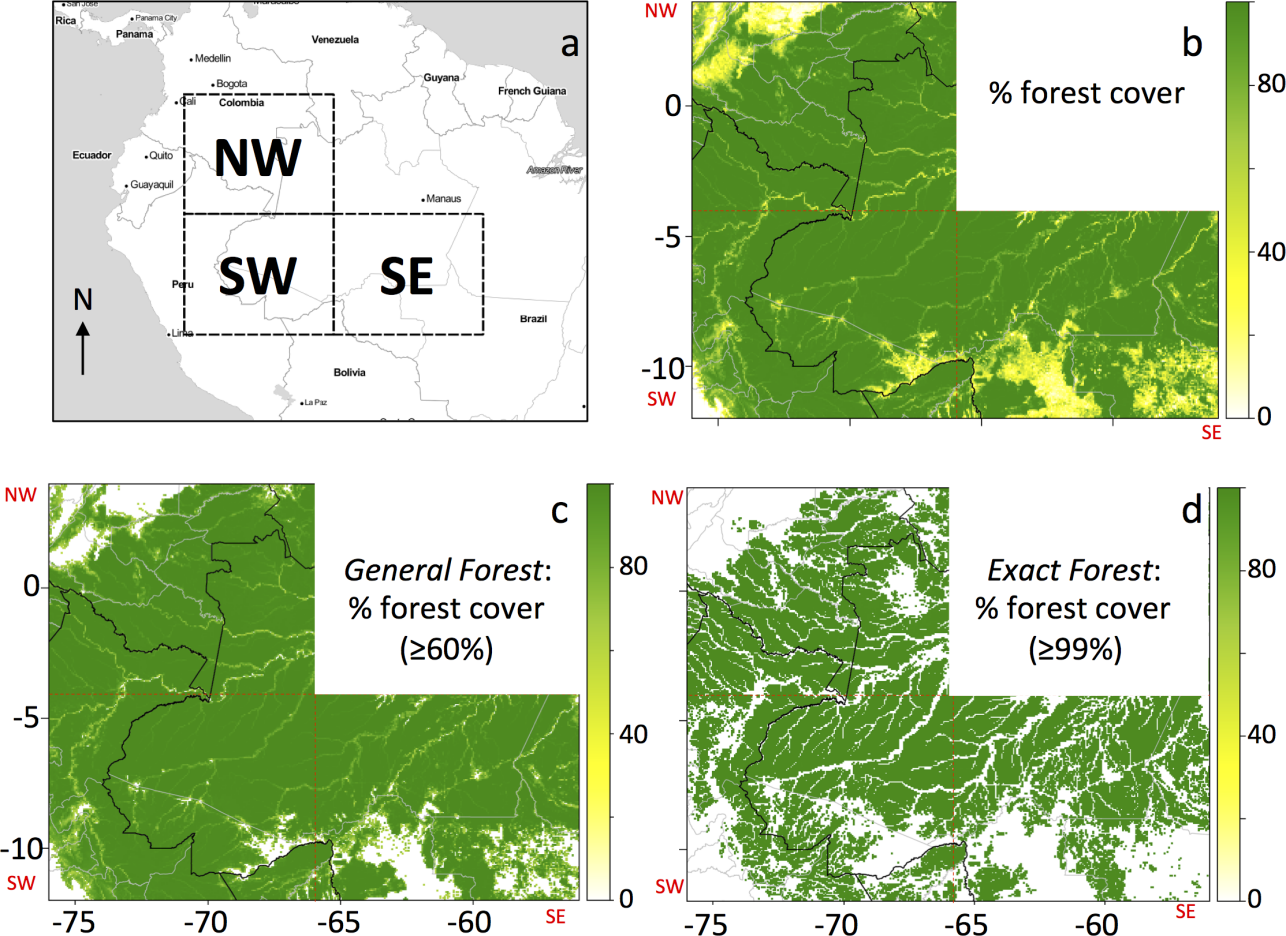
Forest cover maps. (**a**) The three 8° x 10° quadrants under consideration in this paper (NW: 4°N– 4°S, 76°W– 66°W; SW: 4°S– 12°S, 76°W– 66°W; and SE: 4°S– 12°S, 66°W– 56°W). Maps of the study region at 0.05° resolution of (**b**) percent forest cover in 2009, (**c**) percent forest cover in 2009 for the *General Forest* mask (tree cover ≥ 60% in 2009), and (**d**) percent forest cover in 2009 for the *Strict Forest* mask (tree cover ≥ 99% in 2009). In panels **b-d**, the SW, SE, and NW quadrants are identified by red dashed lines; national borders are black lines, state borders are grey lines.

Our area of study includes the 2005 drought-affected region (4°S– 12°S, 66°W– 76°W), as well as two neighboring regions of equivalent size: one to the east (4°S– 12°S, 56°W– 66°W), which had nearly twice as much forest cover loss 2001–2009 [23] and did not experience a severe drought in 2005; and one to the north (4°N– 4°S, 66°W– 76°W), which had only about half as much forest cover loss 2001–2009 [23] and did not experience a drought in 2005. We refer to these areas as the SW, SE, and NW quadrants, respectively (Fig 1A). Each quadrant contains 32,000 grid cells at 0.05°x0.05° spatial resolution and 2,000 cells at the 0.2°x0.2° spatial resolution.

### Forest cover and forest cover change

Hansen et al. [23] mapped global forest cover percent in 2000, annual forest cover loss from 2001–2013, and percent water cover in 2000 (assumed static). These products were derived from Landsat and are available at approximately 30-m resolution (1 arc-sec). We aggregated these data for our three study regions to 0.05° and 0.2° to create maps of percent forest cover in 2000, annual forest loss for 2001–2009 (temporal overlap with Qscat data), and percent cover in 2010 (percent cover in 2000 minus total loss 2001–2009) (Fig 1B). We also computed the distance from each 0.05° grid cell to the nearest grid cell with <30% forest cover, as a metric of proximity to land use and human activity, e.g., cleared land or major rivers.

Using these aggregated forest cover data, we produced two forest cover masks for each of the three regions: (1) ‘*General Forest’* grid cells that had forest cover in 2010 ≥ 60% (Fig 1C); and (2) ‘*Strict Forest’* grid cells had forest cover in 2010 ≥ 99% (Fig 1D). We chose a 60% threshold for the *General Forest* because that is the threshold for the MODIS forest land cover classification [24, 25], and was the threshold used by Saatchi et al. [18] for the MODIS vegetation continuous fields (VCF) forest mask. We consider the *General Forest* mask to be very similar, though not identical, to the forest mask used by Saatchi et al. [18].

### Metrics for the 2005 Amazon drought

**Fig 2:**
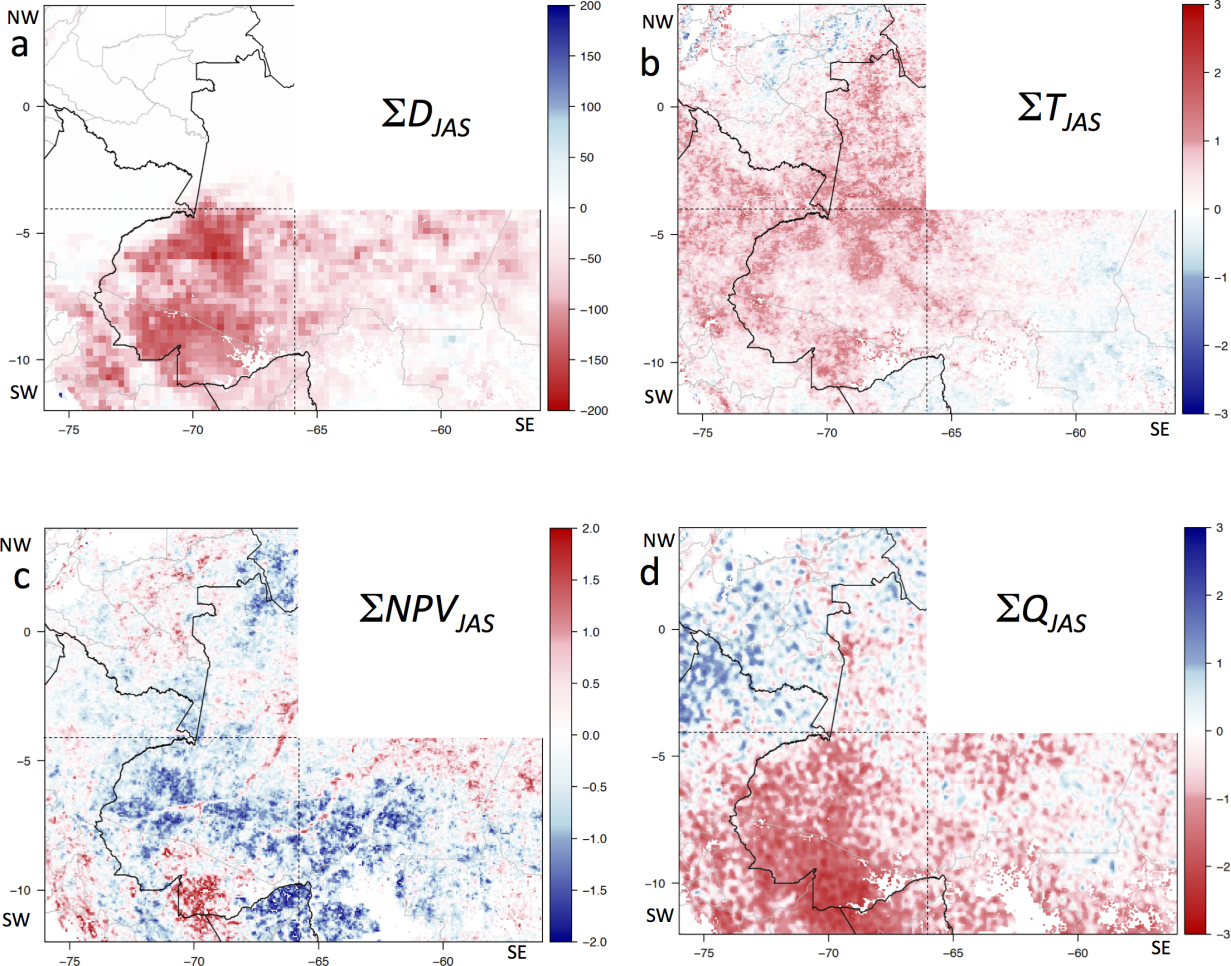
Direct drought impact maps. Maps of variables during the 2005 drought during July-August-September (JAS), using the *General Forest* mask (see Fig 1C). (**a**) ∑*D*_*JAS*_, the total JAS 2005 TRMM cumulative precipitation deficit anomaly (mm); (**b**) ∑*T*_*JAS*_, sum of JAS 2005 MODIS AQUA LST anomalies (°C); (**c**) ∑*NPV*_*JAS*_, mean JAS 2005 MODIS NPV normalized anomaly; and (**d**) ∑*Q*_*JAS*_, mean JAS 2005 QScat normalized backscatter power anomaly. Backscatter power: *Q* = 10^(sig^0^/10), where sig^0^ is the backscatter in dB. The SW, SE, and NW quadrants are identified by black dashed lines; national borders are solid black lines, state borders are grey lines.

We used four remote sensing data sets to map and quantify the extent and direct impact of the drought on the forest (see Table 1 for details): (i) TRMM mean dry season 2005 accumulated monthly precipitation deficit anomaly (∑*D*_*JAS*_) as a measure of the strength of the drought, based on a 100 mm/month precipitation threshold [7, 26, 27] (Fig 2A); (ii) mean dry season 2005 MODIS AQUA daytime (overpass c.13:30 local time) land surface temperature anomaly (∑*T*_*JAS*_) (Fig 2B)–a drier canopy will be warmer, as shown for this drought [28]; (iii) mean dry season 2005 MODIS non-photosynthetic vegetation (NPV) cover anomaly (∑*NPV*_*JAS*_) (Fig 2C); MODIS NPV was generated using Multiple Endmember Spectral Mixture Analysis (MESMA: [29]), and should be sensitive to any substantial loss of canopy foliage due to the drought; and (iv) Qscat mean dry season (July, August, and September, or JAS) 2005 normalized anomaly (∑*Q*_*JAS*_), which has been shown to register a strong negative anomaly in morning overpass data during the drought [18, 19] (Fig 2D).

**Table 1:** Variables used in the study.

Variable	Description	Equation	Data source
*D*_*t*_	Monthly accumulating precipitation deficit (mm)	*D*_*t*_ = min[0, *D*_*t-1*_ + (*P*– 100)]	*P*–monthly precipitation (mm) from TRMM 3B43-v6, for month *t;* downscaled to 0.05° resolution by triangle-based linear interpolation.
*D*_t_*	Monthly accumulating precipitation deficit anomaly (mm).	*D*_t_* = *D*_*t*,*2005*_ –mean_*2001-09*_(*D*_*t*_)	*See above*. *Note*: *if wet season does not fully offset dry season deficit*, *i*.*e*., Dt* ≠ 0 at end of wet season, *grid cell is excluded*.
∑*D*_*JAS*_	Sum of D* for JAS 2005 (dry season of drought year) (mm).	∑*D*_*JAS*_ *= D*_July_* + D_Aug_* + D_Sept_*	*See above*.
Δ*D*	Post- minus pre-drought mean accumulating precipitation deficit anomaly (mm).	Δ*D =* mean_*2006-09*_(∑*D*_*JAS*_)*–*mean_*2001-04*_(∑*D*_*JAS*_)	*See above*.
*T*_t_*	Monthly land surface temperature anomaly (°C).	*T*_t_* = *T*_*t*,*2005*_ –mean_*2002-09*_(*T*_*t*_*)*	*T*_*t*_−monthly Land Surface Temperature (°C) from MODIS c.5 MYD1C11, for month *t*, using QA flag = 0 or 65, with ‘good quality data’ and ‘average emissivity error < = 0.01’.
∑*T*_*JAS*_	Sum of T* for JAS 2005 (dry season of drought year) (°C).	∑*T*_*JAS*_ *= T*_July_* + T_Aug_* + T_Sept_*	*See above*.
*T*	Post- minus pre-drought mean land surface temperature.	Δ*T =* mean_*2006-09*_(∑*T*_*JAS*_)–mean_*2002-04*_(∑*T*_*JAS*_)	*See above*.
*Q*_t_*	Monthly normalized QScat backscatter power return anomaly.	*Q*_t_* = (*Q*_*t*,*2005*_ –mean_*2001-09*_(*Q*_*t*_)) ÷ std.dev_*2001-09*_(*Q*_*t*_)	*Q*_*t*_ *= 10^(*sig^0^*/10);* QScat quev sig^0^ from www.scp.byu.edu.
∑*Q*_*JAS*_	Sum of Q* for JAS 2005 (dry season of drought year).	∑*Q*_*JAS*_ *= Q*_July_* + Q_Aug_* + Q_Sept_*	*See above*.
Δ*Q*	Post- minus pre-drought mean normalized QScat backscatter.	Δ*Q =* mean_*2006-09*_(∑*Q*_*JAS*_)*–*mean_*2001-04*_(∑*Q*_*JAS*_)	*See above*.
Δ*LC*	Forest cover loss 2005–09.	Δ*LC =* ∑_*2005-09*_*(FC*_*loss*_*)*	*FC*_*loss*_ is annual gross forest cover loss as percent of grid cell area [23].
*Z*_*F30*_	Grid cell distance (km) from nearest grid cell with forest cover ≤30% in 2000.	Computed in ArcGIS.	[23]
*NPV*_t_*	Monthly normalized non-photosynthetic vegetation fraction anomaly.	*NPV*_t_* = (*NPV*_*t*,*2005*_ –mean_*2001-09*_(*NPV*_*t*_)) ÷ std.dev_*2001-09*_(*NPV*_*t*_)	MODIS c.5 MCD43A4 product converted to fractions of green vegetation, non-photosynthetic vegetation (NPV) and shade using spectral mixture analysis.
∑*NPV*_*JAS*_	Sum of NPV* for JAS 2005 (dry season of drought year).	∑*NPV*_*JAS*_ *= NPV*_July_* + NPV_Aug_* + NPV_Sept_*	*See above*.

Variables were computed for each 0.05° grid cell in all quadrants. Subscript *t* refers to time by month.

### Metrics for post-drought change/persistence

**Fig 3:**
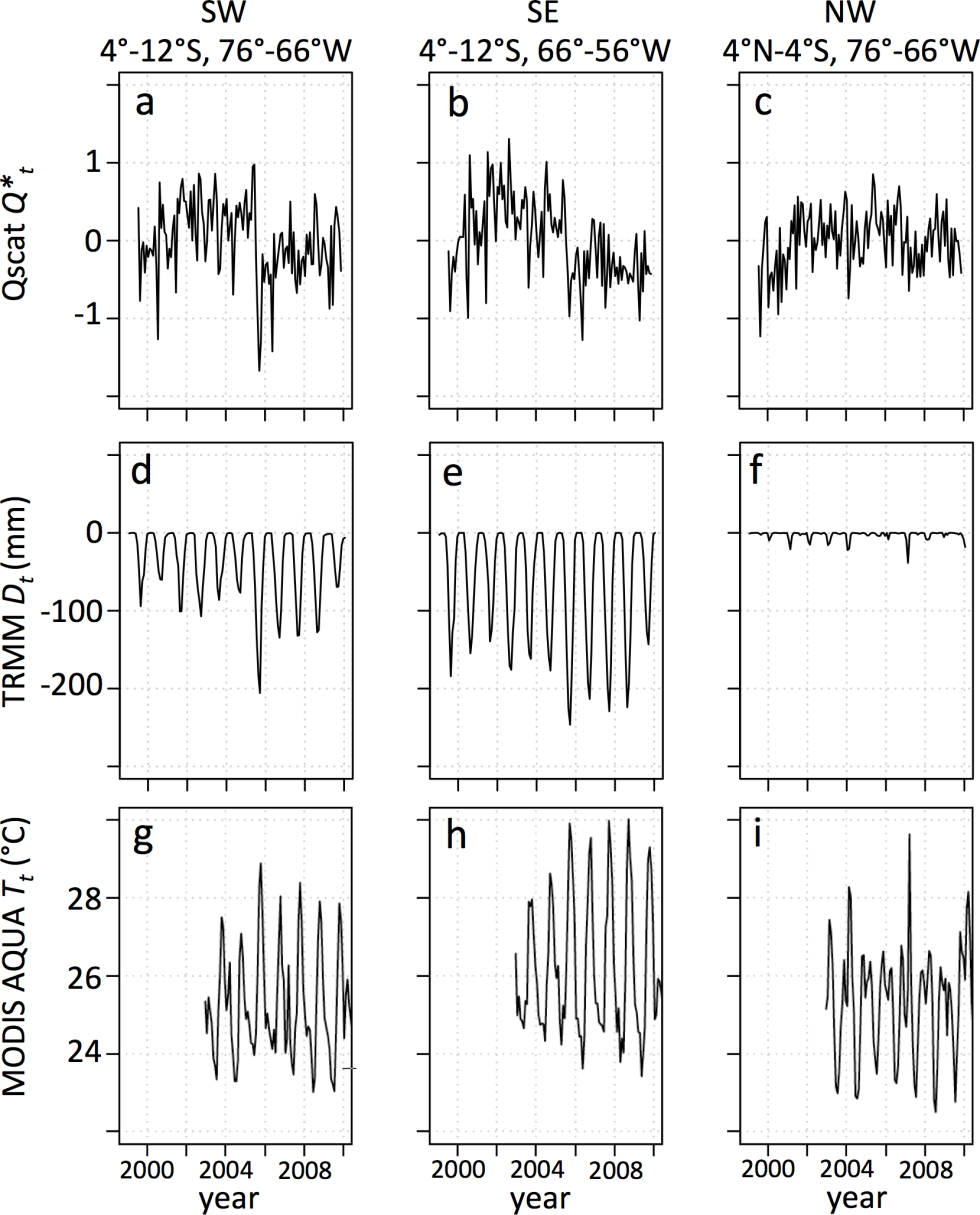
Regional drought variable time series. Mean monthly QScat normalized backscatter power anomaly (Q*_t_) aggregated over the *General Forest* mask for the (**a**) SW, (**b**) SE, and (**c**) NW quadrants (see map in Fig 1A). Mean monthly TRMM cumulative precipitation deficit (*D*_*t*_) over the *General Forest* mask for the (**d**) SW, (**e**) SE, and (**f**) NW quadrants. Mean monthly MODIS AQUA LST (*T*_*t*_) aggregated over the *General Forest* mask for the (**g**) SW, (**h**) SE, and (**i**) NW quadrants. Backscatter power: *Q* = 10^(sig^0^/10), where sig^0^ is the backscatter in dB.

**Fig 4:**
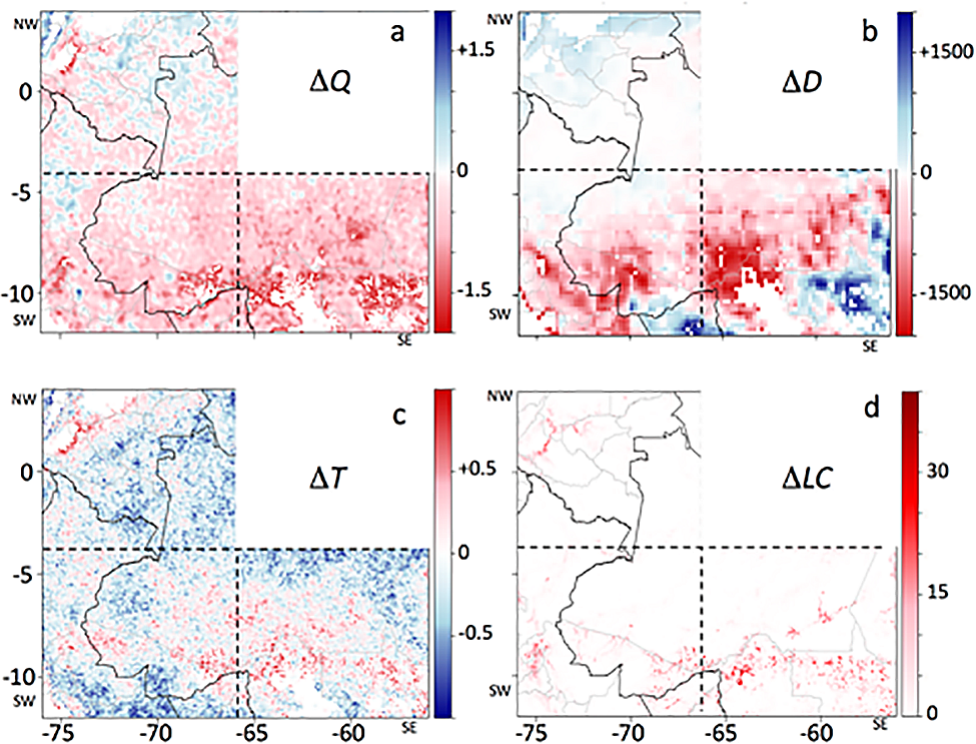
Maps of pre- to post-drought changes. Maps, using the *General Forest* mask (see Fig 1), of variable changes post-2005 drought (2006–2009) minus pre-2005 drought (2001–2004): (**a**) Δ*Q*, (**b**) Δ*D*, (**c**) Δ*T*, and (**d**) Δ*LC* (see Table 1 for variable definitions). Backscatter power *Q* = 10^(sig^0^/10), where sig^0^ is the backscatter in dB. The SW, SE, and NW quadrants are identified by black dashed lines; national borders are solid black lines, state borders are grey lines.

We computed three metrics of the difference between pre-drought (2001–2004) and post-drought (2006–2009) vegetation and physical environment changes (see Table 1), using monthly time series derived from Qscat, TRMM, and MODIS LST data, which all show a change in values in the SW & SE quadrants after the drought, but not in the NW quadrant (Fig 3). These computed post-drought minus pre-drought differences were mapped at 0.05° resolution (Fig 4). One of these, the dry season Qscat backscatter (Fig 4A), is the key variable of interest, because it is a measure of the persistent drop in backscatter that manifested for the forested SW region. We also computed and mapped changes in dry-season precipitation deficit (Fig 4B), and post-drought minus pre-drought mean dry season MODIS land surface temperature (Fig 4C). Note that MODIS Aqua data collection began in May 2002, so the pre-drought period was only 3 years, i.e., 2002–2004 rather than 2001–2004. We also computed forest cover loss 2005–2009 as a percent of each 0.05° grid cell from the global forest cover product [23] (Fig 4D).

### Analyses

We generated maps of key variables in an effort to visually examine spatial patterns in the data. Furthermore, we used these key variables in a series of statistical analyses in an attempt to ascribe environmental processes to drought impacts that were evident in the remotely sensed data. We developed multiple linear regression models to quantify correlation strength across the region between our chosen dependent variable, i.e., persistent post-drought low QScat backscatter (ΔQ), and variables quantifying drought magnitude, drought impact on the forest canopy, and persistent changes in weather. Multiple linear regression analyses were conducted at two spatial resolutions, first at 0.05°, using Delaunay triangular interpolation of the 4.45-km composited QScat data provided by the NASA Scatterometer Record Pathfinder (SCP) project ([30]; http://www.scp.byu.edu/), and then aggregated to 0.2°, which is closer to the instrument native sensor resolution of ~25 km [30] to see if that improved the correlations. Then, using the 0.2° aggregated data, which had stronger correlations (see Results section below), we conducted a heuristic classification of the region by identifying grid cells with ‘extreme’ variable magnitudes, i.e., greater than one standard deviation from a regional mean, to assess whether any patterns that were apparent from visual inspection were supported by evaluating the co-occurrence of moderate to strong anomalies. Finally, we tested a hypothesis that a simple attribution model could explain the occurrence and strength of low ΔQ values.

#### Spatial patterns

We mapped the magnitudes of several classes of variables (see Table 1) at 0.05° resolution (~5 km): (i) 2005 drought exposure: JAS 2005 precipitation deficit (∑*D*_*JAS*_ Fig 2A); (ii) immediate vegetation sensitivity to drought: JAS 2005 land-surface temperature anomalies (∑*T*_*JAS*_, Fig 2B), JAS 2005 NPV anomaly (∑*NPV*_*JAS*_, Fig 2C), and JAS 2005 Qscat mean anomaly (∑*Q*_*JAS*_, Fig 2D); (iii) precipitation deficit and land-surface temperature (LST) change pre- to post-drought (Δ*D*, Fig 4B*;* Δ*T*, Fig 4C); and (iv) canopy change: total forest cover loss 2005–2009 (Δ*LC*, Fig 4D) and backscatter change pre- to post-drought (Δ*Q*, Fig 4A).

#### Classification analysis

We then aggregated and aligned the data described above to a 0.2 x 0.2° (~20 km) grid, and using the distribution of values for *General Forest* grid cells in the SW quadrant at 0.2°, we developed thresholds based on the statistical distribution of each of the variables for drought exposure (*D*_*i*_), canopy sensitivity (*Q*_*i*_), forest cover loss 2005–2009 (Δ*LC*_*i*_), precipitation change (Δ*D*_*i*_), and persistent backscatter drop (Δ*Q*_*i*_). Using the thresholds, we developed binary classifications for each grid cell for each of these metrics, e.g., drought exposure (*D*_*i*_) is ‘true’ if JAS 2005 mean precipitation deficit normalized anomaly (or Z-score) was less than -1, otherwise it is ‘false’ (we also tested alternative cases, with Z-score thresholds at -1.5 or -0.67). Each grid cell was classified based on which of the 4 independent variable metrics exceeded the threshold (true or false for 4 metrics gives 16 possible classes). We then determined the number of grid cells in each of the classes that had a persistent backscatter drop that exceeded the threshold, and presented these as a proportion of all grid cells. We used threshold metrics determined from data for the SW quadrant to analyze grid cells in all three quadrants under consideration (SW, NW, SE).

#### Statistical models

We developed multiple linear regression models to quantify correlations across the domain (using 0.05° grid cells, index *i*) between a suite of independent variables, i.e., JAS 2005 drought metrics (DR: ∑*D*_*i*_, ∑*T*_*i*_, ∑*Q*_*i*_, ∑*NPV*_*i*_), land cover metrics (LC: Δ*LC*_*i*_*; Z*_*F30*,*i*_), precipitation and LST change metrics (WE: Δ*D*_*i*_*;* Δ*T*_*i*_*)*, and the dependent variable, i.e., persistent post-drought drop in Qscat backscatter (Δ*Q*_*i*_). The multiple linear regression models aggregated independent variables into drought metrics (DR), land cover change metrics (LC) and precipitation and LST change metrics (WE) and combinations of these (LC & DR, LC & WE, DR & WE, LC & DR & WE).

We tested three additional spatial variables–grid cell elevation from GTOPO30 [31], presence of significant bamboo vegetation cover [32, 33], and annual area burned in understory fires (MODIS burned area product MCD64A1 [34])–in simple and multiple linear regression analyses against the dependent variable Δ*Q*. They all had insignificant correlations (R^2^ ≤ 0.01), and no further results are presented.

#### Attribution model

Finally, we evaluated the hypothesis that the Qscat monthly normalized backscatter anomaly time series for each grid cell could be explained as a superposition of two signals: (1) a linear decline in backscatter representing gradual loss of forest cover at 0.2°x0.2°, and (2) a drop in backscatter at the time of the 2005 drought followed by a linear recovery. We therefore fit a piecewise linear ‘attribution model’ for the QScat backscatter power monthly anomalies (Q*) against time (*t*).

$$\begin{array}{l} Q^{*}(t)=m_{L}\cdot t+Q_{0}\;t<\mathrm{Jan}.\;2005\\ Q^{*}(t)={\left(m_{L}+m_{d1}\right)}_{L}\cdot t+Q_{0}\;\mathrm{Jan}.\;2005\leq t\leq \mathrm{Dec}.\;2005\\ Q^{*}(t)={\left(m_{L}+m_{d2}\right)}_{L}\cdot t+Q_{0}\;t>\mathrm{Dec}.\;2005 \end{array}$$ (1)

where *m*_*L*_ represents the slope of the land cover change trend, *m*_*d1*_ is the slope of the initial response to the drought, i.e. any QScat change that occurred during 2005, *m*_*d2*_ is the slope of the post-drought response, and *Q*_*0*_ is a constant offset to get an overall expected mean Q* value of zero anomaly. For a cell to be consistent with our hypothesis, *m*_*L*_ and *m*_*d1*_ would be negative, and *m*_*d2*_ would be positive (assuming a post-drought recovery). The explanatory power of the model for each of the 0.2° grid cells across the domain was also recorded as percent variance explained (R^2^).

## Results

### Spatial patterns

The western Amazon is predominantly covered by forest (Fig 1B). Roughly 90% of the 0.05° grid cells in the region fall in the *General Forest* mask category (forest cover ≥ 60% in 2010), with 95% in the SW quadrant, 85% in the SE quadrant, and 91% in the NW quadrant (Fig 1C). Roughly 55% of the 0.05° grid cells in the region fall in the more restrictive *Strict Forest* mask category (forest cover ≥ 99% in 2010), with 57% in the SW quadrant, 51% in the SE quadrant, and 59% in the NW quadrant (Fig 1D).

As previously noted [18], a persistent drop in QScat backscatter followed the strong backscatter anomaly during the 2005 drought [19] in a large portion of the SW Amazon forest region (4°-12°S, 76°-66°W). A similar persistent drop in backscatter during 2006–2009 was widespread to the east of this region (4°-12°S, 66°-56°W) (Fig 4A), although this SE box did not have a strong 2005 backscatter anomaly (Fig 3B). In the northwest Amazon forest region, there was no widespread persistent drop in backscatter during 2006–2009, nor can a 2005 drought backscatter anomaly be observed (Figs 4A and 2D), providing additional evidence that the sensor degradation does not explain the persistent differences observed elsewhere.

The TRMM data show that the precipitation deficit during 2005 in the SW Amazon forest region was anomalously strong (Fig 2A), and that the subsequent three dry seasons were drier than the six pre-drought dry seasons (Fig 3D). Recent dry seasons have been generally longer and drier to the east of this region (SE quadrant), and here also the 2005–2008 dry seasons were drier than the previous six years (Fig 3E). The northwest Amazon does not exhibit a significant precipitation deficit (Fig 3F). Similarly, dry-season MODIS AQUA daytime LST was higher after the 2005 drought in the SW quadrant, and particularly in the SE quadrant, but not in the NW quadrant (Fig 3G–3I).

The 2005 drought was centered in the southwestern Amazon, while the SE quadrant of our study region was also somewhat drier than normal, but the NW quadrant was not (Fig 2A). Drought impact during July-Sept. 2005 was evident in warmer than normal MODIS LST in the SW (Fig 2B; [29]). The NW quadrant also had higher LST than normal. There was a negative NPV anomaly during July-Sept. 2005 throughout parts of the SW and SE quadrants, and a strong positive NPV anomaly c.10°S, 70°W (Fig 2C). Finally, there was a strong QScat backscatter anomaly during July-Sept 2005 in the SW quadrant (Figs 2D and 3A), though this was only observed in the morning overpass data [19].

The persistent, post-drought drop in July-Sept. morning backscatter anomaly occurred across essentially all of the SW and SE quadrants, and frequently, though not always, was strongest in forested grid cells adjacent to those cells excluded by the *General Forest* mask (Fig 4A). The post-drought (2006–2009) JAS dry seasons had greater precipitation deficits than the pre-drought (2001–2004) JAS dry seasons throughout much of the SW and SE quadrants (Fig 4B). LST was higher post-drought in many, but not all, of the regions that were significantly drier post-drought (compare Fig 4C and 4B), but generally lower outside of those regions (Fig 4C). Forest cover loss in *General Forest* grid cells (which still had >60% forest cover in 2010) occurred across the southern portions of the SW and SE quadrants, and in the northwest corner of the NW quadrant (Fig 4D).

**Fig 5:**
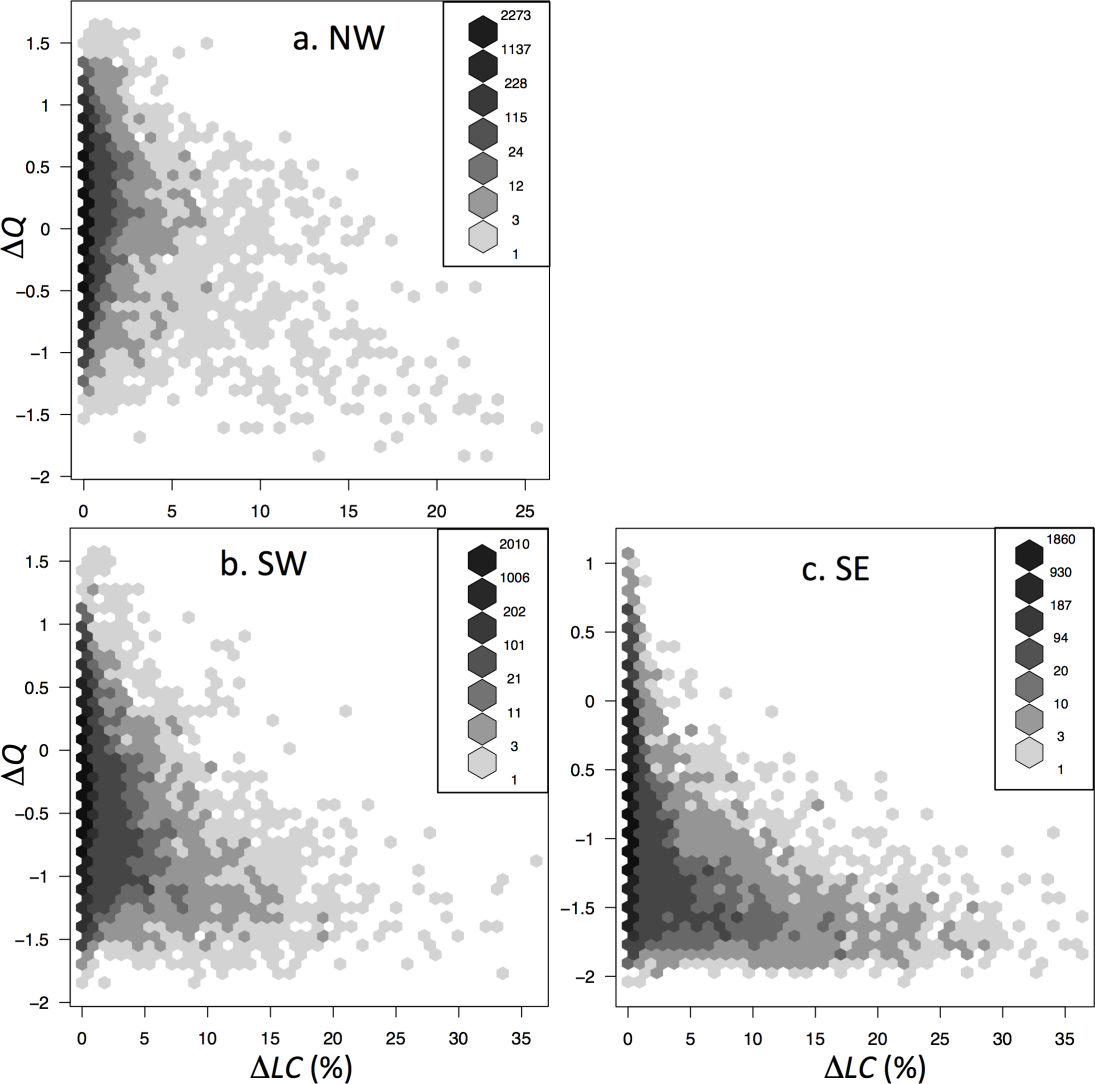
Persistent backscatter drop versus land cover change. Binned scatterplots of post-drought JAS (2006–2009) mean QScat normalized backscatter power anomaly minus pre-drought JAS (2001–2004) mean QScat normalized backscatter power anomaly (Δ*Q*) vs. percent forest cover loss (ΔLC) during 2005–2009 ([23]; see Fig 4D) for 0.05° grid cells and *General Forest* mask in the (**a**) NW, (**b**) SW, and (**c**) SE quadrants (see Fig 1). Note that the axis range varies between panels. The color bars are grid cell number density per bin. Backscatter power: *Q* = 10^(sig^0^/10), where sig^0^ is the backscatter in dB.

*General Forest* grid cells by definition had ≥ 60% forest cover in 2010, but could have lost forest cover during 2001–2009 if they had high forest cover in 2000. *General Forest* grid cells that lost more than about 15% (SW) or 10% (SE) of their forest cover during 2005–2009 (drought year and post-drought) consistently showed a persistent drop in QScat backscatter post-drought (Fig 5). This pattern was observed to be weaker in the NW quadrant (Fig 5).

### Statistical models

Overall, at 0.05° resolution, goodness-of-fit metrics across the *General Forest* domain of the various drought, land cover, precipitation and LST metrics as predictors of the persistent drop in QScat backscatter were moderate (R^2^ between approximately 0.2 and 0.4, mostly in the SE quadrant) to weak (R^2^ < 0.2, mostly in the SW and NW quadrants) (Table 2). In the SW quadrant, where the 2005 drought occurred, land-cover change and drought were stronger predictors of persistent backscatter drop than was post-/pre-drought precipitation and LST differences. In the SE quadrant, the stronger predictors were land cover change and post-/pre-drought precipitation and LST differences, while in the NW no variables were found to be even moderate predictors. Aggregating the data to 0.2° resolution increased the goodness-of-fit metrics in essentially all cases (Table 2). However, only for the SE quadrant *Strict Forest* domain case were goodness-of-fit metrics at 0.2° resolution greater than 0.4, with only drought impact (DR) below that value (R^2^ = 0.14). As would be expected, restricting the analysis to the *Strict Forest* domain eliminated any predictive power of land cover change, as the *Strict* Forest had negligible forest cover loss during 2001–2009, and aggregation to 0.2° resolution did not improve those metrics (Table 2). Given the improved metrics of the 0.2° analysis, the classification and attribution analyses were done at this resolution.

**Table 2:** Multiple linear regression results.

Forest/Region^a^	# cells (%)	LC^b^	DR^b^	WE^b^	LC & DR	LC & WE	DR & WE	LC & DR & WE
**0.05° grid cells**								
***General Forest***								
**SW**	30449 (95%)	0.13	0.10	0.04	0.21	0.15	0.15	0.23
**SE**	27258 (85%)	0.23	0.10	0.23	0.29	0.34	0.29	0.37
**NW**	29147 (91%)	0.07	0.04	0.08	0.11	0.16	0.11	0.18
***Strict Forest***								
**SW**	18239 (57%)	0.01	0.10	0.08	0.10	0.09	0.16	0.16
**SE**	16356 (51%)	0.01	0.10	0.14	0.10	0.16	0.21	0.22
**NW**	1886 (59%)	0.00	0.05	0.03	0.06	0.04	0.07	0.08
**0.2° grid cells**								
***General Forest***								
**SW**	1917 (96%)	0.24	0.12	0.07	0.32	0.26	0.23	0.36
**SE**	1746 (87%)	0.45	0.14	0.44	0.51	0.57	0.49	0.60
**NW**	1831 (92%)	0.12	0.08	0.12	0.20	0.24	0.16	0.27
***Strict Forest***								
**SW**	849 (42%)	0.01	0.12	0.15	0.12	0.15	0.22	0.22
**SE**	656 (33%)	0.00	0.16	0.24	0.16	0.25	0.31	0.31
**NW**	847 (42%)	0.00	0.09	0.04	0.10	0.05	0.11	0.13

Multiple linear regression model correlation coefficients, R^2^_ajd_, for 0.05° grid cells and for 0.2° grid cells. Dependent variable is Δ*Q*, independent variables, grouped into categories of land-cover change (LC), drought impact (DR), and weather change (WE), are listed in footnote (see Table 1 for variable details).

^a^ *Region extents*: **SW**: 4°S—12°S, 76°W—66°W; **SE**: 4°S—12°S, 66°W—56°W; **NW:** 4°N—4°S, 76°W—66°W.

^b^ *Regression independent variables*: **LC**: ∆*LC* & *Z*_*F30*_; **DR:** ∑*D*_*JAS*_ & ∑*T*_*JAS*_ & ∑*Q*_*JAS*_ & ∑*NPV*_*JAS*_; **WE:** ∆*D* & ∆*T*.

### Classification analysis

Using the SW quadrant *General Forest* data as the basis for setting distribution thresholds, and using a 1-sigma threshold (1 standard deviation) for ‘significance’, the majority of forested grid cells in the the SW quadrant (1175 out of 1917, or about 60%) were in Class 0, and had no significant drought (∑*D*_*JAS*_), drought sensitivity (∑*Q*_*JAS*_), land cover change (Δ*LC*), or pre- to post-drought precipitation deficit change (Δ*D*) (Table 3). The other 742 *General Forest* grid cells in the SW quadrant (~40%) had one or more of these at the 1-sigma level on the dry/disturbed tail, and were in Classes 1–15 (Table 3). This is consistent with statistical expectations if distributions are roughly normal, as the non-affected fraction falls between what would occur with normal distributions with completely coincident drivers (84% with no significant values for Δ*LC*, Δ*D*, ∑*Q*_*JAS*_, ∑*D*_*JAS*_) and no overlap in drivers (36% with none). This result indicates that there is moderate interaction in drivers in the SW quadrant (also see Fig 2).

**Table 3:** Classification analysis results.

					SW		NW		SE	
Class	∑*D*_*JAS*_	∆*LC*	∆*D*	∑*Q*_*JAS*_	N_cells_	∆*Q*	N_cells_	∆*Q*	N_cells_	∆*Q*
0	0	0	0	0	1175	116	1776	22	1285	737
1	1	0	0	0	220	18	1	0	1	0
2	0	1	0	0	106	65	53	10	188	180
3	1	1	0	0	5	3	0	0	0	0
4	0	0	1	0	60	22	0	0	178	158
5	1	0	1	0	13	3	0	0	0	0
6	0	1	1	0	18	11	0	0	87	87
7	1	1	1	0	3	2	0	0	0	0
8	0	0	0	1	155	9	1	0	2	2
9	1	0	0	1	36	9	0	0	0	0
10	0	1	0	1	32	14	0	0	5	5
11	1	1	0	1	6	4	0	0	0	0
12	0	0	1	1	53	12	0	0	0	0
13	1	0	1	1	27	13	0	0	0	0
14	0	1	1	1	6	5	0	0	0	0
15	1	1	1	1	2	1	0	0	0	0
***TOTAL***				*** ***	***1917***	***307***	***1831***	***32***	***1746***	***1169***
Any one or more drivers (Classes 1–15)					741	191	55	10	461	432
at least ∑***D***_***JAS***_ (Classes 1, 3, 5, 7, 9, 11, 13, 15)					312	53	1	0	1	0
at least ∆***LC*** (Classes 2, 3, 6, 7, 10, 11, 14, 15)					178	105	53	10	280	272
at least ∆***D*** (Classes 4–7, 12–15)					182	69	0	0	265	245
at least ∑***Q***_***JAS***_ (Classes 8–15)					317	67	1	0	7	7

*General forest* grid cell (0.20°) analysis to determine co-locations in the SW, NW, and SE quadrants (see Fig 1) of Qscat post-drought persistent low backscatter (Δ*Q*) with classification by 2005 drought precipitation deficit (∑*D*_*JAS*_), land cover change 2005–2009 (Δ*LC*), drought QScat sensitivity (∑*Q*_*JAS*_), and pre- to post-drought precipitation deficit change (Δ*D*), using a 1-sigma threshold based on the distributions of ∑*D*_*JAS*_, Δ*D*, ∑*Q*_*JAS*_, and Δ*LC* in the *General Forest* in the SW quadrant (see Figs 1–4). Quadrant values are number of grid cells in each class (N_cells_) and number of those grid cells with Δ*Q* more than one standard deviation on the dry/disturbed end of the distribution. See variable definitions in Table 1.

Ten percent of the Class 0 *General Forest* grid cells in the SW quadrant had a significant post-drought low backscatter (Δ*Q*), while sixteen percent of the grid cells in Classes 1–15 had a significant post-drought low backscatter (Δ*Q*) (Table 3), indicating that low backscatter during 2006–2009 was somewhat more likely for grid cells that experienced drought or lost forest cover or had drier weather. Of the grid cells that experienced significant (>1σ) anomalies in one or more of the dry/disturbed conditions we evaluated, ΔLC was the strongest predictor of low 2006–2009 backscatter (105 out of 178 grid cells or 59%), while ∑*D*_*JAS*_ was the weakest predictor (53 out 312 grid cells or 17%), and strong backscatter sensitivity during the drought (∑*Q*_*JAS*_) was only a slightly stronger predictor than that (67 out 317 grid cells or 21%). All of these general patterns remained similar when the significance threshold values for each variable were raised to 1.5-sigma and lowered to 0.67-sigma.

In the NW quadrant, almost all grid cells (97%) were in Class 0 with no significant values of any of the factors, based on SW quadrant distribution thresholds (Table 3). This is consistent with a minimal dry season, no 2005 drought, and relatively low land use pressure in this quadrant (see Figs 2–4). In the SE quadrant, to the east of the 2005 drought, again the majority of grid cells (74%) were in Class 0, and had no significant drought (∑*D*_*JAS*_), backscatter drought sensitivity (∑*Q*_*JAS*_), land cover change (Δ*LC*), or pre- to post-drought precipitation change (Δ*D*). However, a much larger fraction of the grid cells in the SE quadrant had a 1-sigma significant persistent post- minus pre-drought drop in backscatter (Δ*Q*) than in the SW quadrant (1169 out of 1746, or 67%) (Table 3). In the SE quadrant, the strongest co-occurrence of low backscatter during 2006–2009 was with Δ*LC* and Δ*D*, although the majority of the SE quadrant grid cells with significant persistent backscatter change were in Class 0, and did not have any significant values of the evaluated factors. These general patterns in the NW and SE quadrants also remained similar when the significance threshold values for each variable were raised to 1.5-sigma and lowered to 0.67-sigma.

### Attribution model

**Fig 6:**
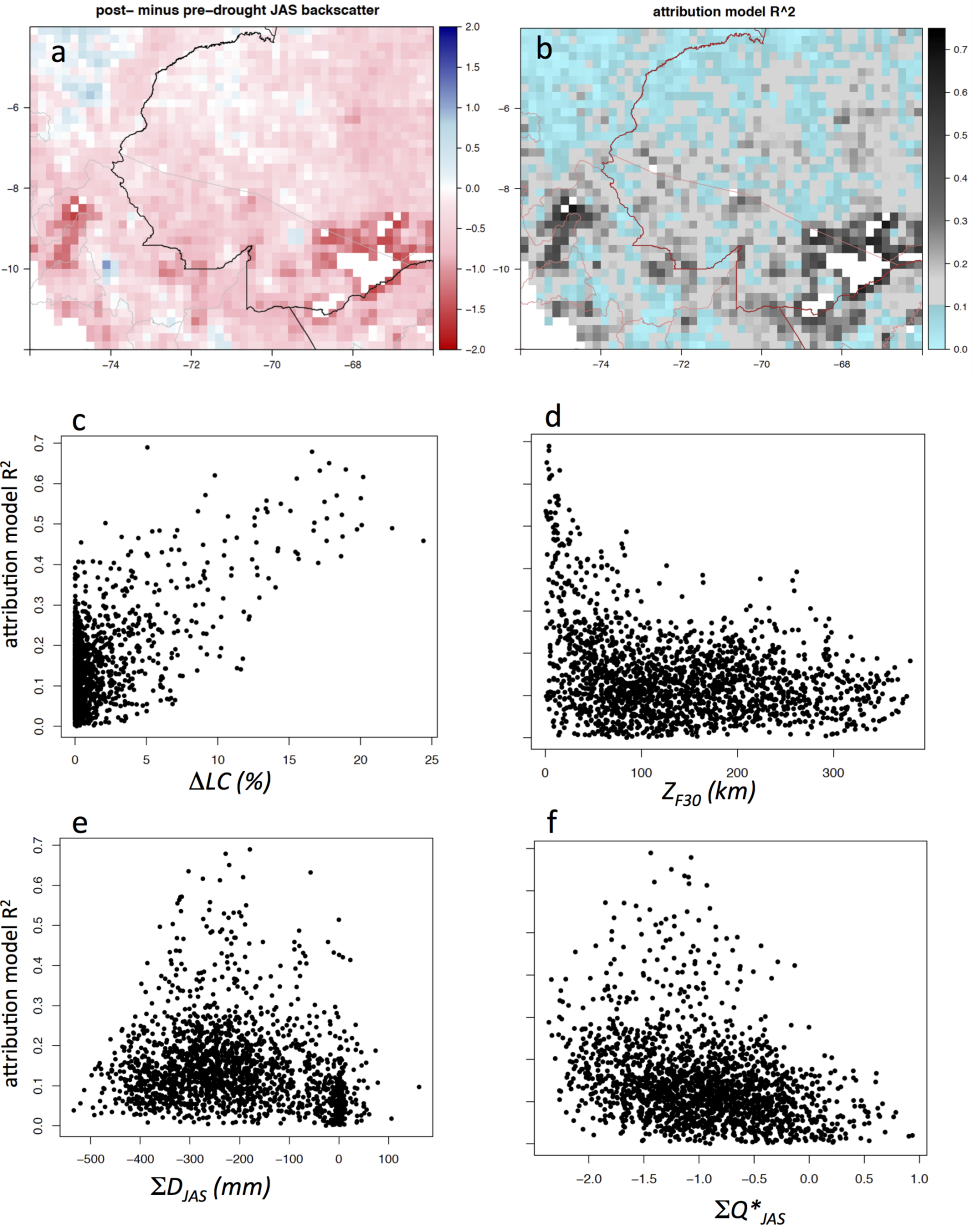
Attribution model summary results. Attribution model (Eq 1) fit to QScat monthly normalized anomaly time series (*Q*_t_*) at 0.2° grid resolution for the SW quadrant (n = 1917); (**a**) the model dependent variable, Δ*Q* (see Table 1), and (**b**) variance explained by the model (R^2^). National borders are black or red lines; state borders are thin grey or pink lines. (**c-f**) Grid cell scatterplots of attribution model correlation vs. (**c**) Δ*D*, (**d**) *Z*_*F30*_, (**e**) ∑*D*_*JAS*_, and (**f**) ∑*Q*_*JAS*_ (see Table 1 for variable definitions).

**Fig 7:**
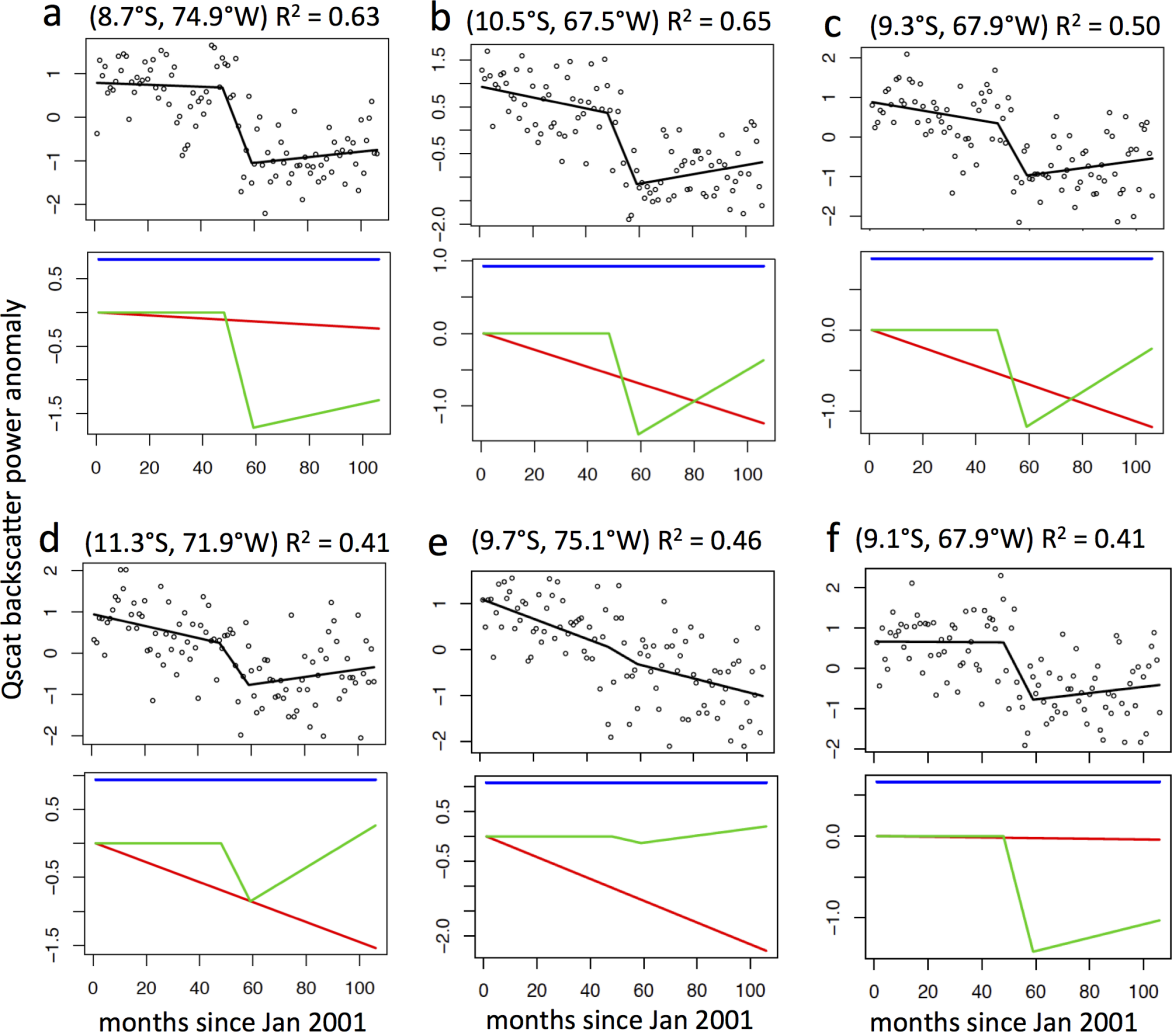
Attribution model sample results. (*upper panels of pairs*) Attribution model (Eq 1) fit to Qscat monthly normalized anomaly time series (*Q*_t_*) for six 0.2° grid cells in the SW quadrant with moderate to good fits (R^2^ > 0.4); (*lower panels of pairs*) the three components of attribution mode: linear land cover change trend (red line: *m*_*L*_ in Eq 1), piecewise linear drought response and recovery (green line: zero until 2005, then *m*_*d1*_ and *m*_*d2*_ in Eq 1), and mean offset (blue line: *Q*_*0*_ in Eq 1).

The signal targeted by the attribution model was the monthly QScat backscatter anomaly time series, aggregated to 0.2°, which across the SW quadrant is dominated by low post-drought (2006–2009) backscatter (Fig 3A), including during the July-Sept. dry season (i.e., Δ*Q*). Δ*Q* was negative (persistent low dry-season backscatter) across much of the SW quadrant, stronger in the southern half, especially in the vicinity of a large area of deforested land surrounding Rio Branco, Acre (9–11°S, 67–69°W) (Fig 6A). The Rio Branco area has had active deforestation during 2005–2009 (Fig 4D), as well as before and after that period [23]. The simple attribution model, which combined a linear 9-year trend (2001–2009) (i.e., slow and persistent land cover or canopy change) with a drought-year (2005) abrupt trend followed by a steady recovery (2006–2009), could explain up to about 70% of the data variance, though for much of the domain the fraction of variance explained was <20% (Fig 6B). The attribution model predicted the most variance in the time series (highest R^2^) where there was the strongest persistent drop in backscatter (Δ*Q*) (Fig 6A and 6B). Strong attribution was generally associated with >10% forest cover loss 2001–2009 (Δ*LC*) (Fig 6C, and see also Fig 5B) and proximity to land with low (<30%) forest cover (*Z*_*F30*_) (Fig 6D). It was associated with a moderate, but not strong, 2005 precipitation deficit (∑*D*_*JAS*_) (Fig 6E), and only moderately so with the QScat JAS 2005 drought anomaly (∑Q*_JAS_) (Fig 6F). Strong attribution (R^2^ ≥ 0.5) can arise from a primarily drought/recovery fit (e.g., Fig 7A) or a mixed land cover change and drought recovery fit (e.g., Fig 7B and 7C). Moderate attribution (R^2^ > 0.4) can arise from a mixed land cover change and drought recovery fit (e.g., Fig 7D), land cover change only (e.g., Fig 7E), or drought recovery only (e.g., Fig 7F).

## Discussion

The 2005 drought in the Amazon forest was widespread, strong and focused in the southwestern Amazon [11]. A less intense drought in 2007 was more dispersed across the basin, but spatially overlapped with the 2005 drought in a region around 4°-6°S, 68°-72°W, within the SW quadrant of our study [6]. In general, the dry seasons of 2006–2008 were also drier than the 1998–2012 normal, particularly 2007 [6], though that year was similar to 2006 and 2008 in the southwest quadrant (Fig 3D). Jiménez-Muñoz et al. [35] reported a significant warming trend for July-September MODIS LST data for the southwestern Amazon Basin 2000–2012, though the trend was weaker and not significant in the ERA-Interim 2000–2012 data. Similarly, we found that TRMM precipitation deficits in the three post-drought dry seasons were greater (drier) than in the 4 pre-drought years, in the SW and SE quadrants (Fig 3D and 3E), and MODIS LST temperatures were higher post-drought (Fig 3G and 3H), indicating drier dry-seasons during 2006–2009 than in the years prior to the 2005 drought. This seasonal dryness could cause a reduction in QScat backscatter during those periods, and thus contribute to the persistent low values observed following the 2005 drought, independent of forest cover loss in the SW and SE quadrants (Fig 5). This overall reduction in post drought backscatter was strongest in the SE quadrant (Fig 5), consistent with drier post-drought weather in the SE than the SW quadrant, and wetter weather in the NW quadrant (Fig 4B), and inconsistent with the pattern of the 2005 drought in the SW and SE quadrants (Fig 2A).

The NPV anomaly in the SW quadrant differs from the other remote sensing drought metrics in having a strong bimodal pattern (Fig 2). The decrease in NPV through much of the SW quadrant during the 2005 drought (Fig 2C) indicates fewer dead leaves and/or exposed branches contributing to canopy reflectance, and may be due to an atypically large loss of dry-season dead leaves from emergent canopy trees during the drought [36]. In the negative NPV anomaly region, spectral mixture analysis results also show a negative shade anomaly, perhaps indicating less canopy shadowing from emergent tree foliage and more complete canopy closure below that, and a positive GV anomaly, indicating more reflectance from green leaves (not shown). The region with a strong positive NPV anomaly (68°-71°W, 9°-12°S) also has the strongest backscatter anomaly during the drought, and is one region with a strong warm surface temperature anomaly (Fig 2). Post drought, this region has persistently low backscatter, but has cooler surface temperatures and little forest cover loss (Fig 4). Unlike the rest of the study domain, this area of the forest has substantial bamboo cover [32, 33], indicating that bamboo-dominated forest canopies respond to and recover from drought differently than other forests in the region. Widespread bamboo forests in the region are associated with particular soil types that are rare across the rest of Brazil [32], and pre-date human settlement [33].

The SW quadrant of the study region is predominantly forested, where ~95% of 0.05° grid cells have >60% forest cover, the threshold for forest classification in the MODIS land cover product (Table 3). However, new, higher resolution forest cover change data [23] shows that only about 55–60% of the region had complete (≥99%) forest cover in 2010 (Table 3), and that there were losses of a few to up to more than 30% of forest cover during 2005–2009 in many grid cells that still had >60% forest cover in 2010 (Figs 4D and 5B). Fractional forest cover loss leads to a reduced Ku-band microwave backscatter in the humid tropics [22]. This fragmented and gradual forest cover loss is likely contributing to the observed persistent low backscatter, such that most *General Forest* grid cells with forest cover loss >10% had a persistent drop in post-drought microwave backscatter (Fig 5B). This conclusion is reinforced by the even stronger signal of persistent lower backscatter in 2006–2009 in the SE quadrant (Fig 4A), which did not experience strong drought in 2005 (Fig 2A), but had larger forest cover losses during 2001–2009 than in SW or NE quadrants (Figs 4D and 5).

Neither the classification analysis nor the statistical analysis identified strong correlations between the spatial patterns of the persistent post-drought drop in Ku-band microwave backscatter and any of the candidate causal variables examined at the 0.05° or 0.2° scale (c. 5–20 km) (Tables 2 and 3). The simple attribution model also did not reveal strong correlation between hypothesized land cover and drought response trajectories and observed QScat across the majority of the SW quadrant. The explained variance was strong where the signal was strong (Fig 6A and 6B), and it was generally, but not always, the result of contributions of both model components–drought/recovery and forest cover loss (Figs 6 and 7). *General Forest* grid cells with <10% forest cover loss during 2005–2009 had a larger persistent post-drought drop in backscatter in the SE quadrant than the SW quadrant (Fig 5B and 5C), inconsistent with the cause of low backscatter during 2006–2009 being persistent impact from the 2005 drought, which occurred in the SW quadrant.

When aggregated across the SW quadrant, the persistently low post-drought dry-season backscatter probably had several causes–drought impact and slow recovery [18], forest cover loss [22], and a string of drier than normal dry seasons post-drought [6]. But the data are noisy (e.g., Fig 7), and generally none of these factors alone or in combination with others, can explain most of the variance in the backscatter data, at least with the extensive set of models applied in our analyses. In the SE quadrant, the persistent low backscatter (Figs 3B, 4A and 5C), which occurred in two-thirds of the grid cells, was only partially explained by forest cover loss or drier weather (Tables 2 and 3). Other factors that may have contributed to the low post-drought dry-season backscatter operate at fine spatial scales (generally <1 km or <<1 km)–e.g., soil properties, tree species composition and species richness, fine-scale topography–but were not included in our analysis. For example, recent work indicates that forest community structure in the Amazon Basin may be influenced by plant domestication from pre-Columbian times [37]. To the degree that dominant domesticated trees in the Amazon Basin are more or less sensitive to drought, this could also be a factor in the persistent low post-drought backscatter signal (Δ*Q*), but more detailed mapping than is currently available would be needed to conduct this analysis. Some basic misalignments between the signals of the variables considered (see Table 1 and Figs 2 and 4) indicate that conducting the analysis at a finer spatial resolution would not strengthen the correlations; this is supported by the improved correlations generated when the data were aggregated from 0.05° to 0.2° (Table 2).

The persistent reduced QScat backscatter after the 2005 drought is extremely unlikely to be an instrument effect, based in part on the fact that there was no equivalent signal in NW quadrant. We are confident that the backscatter anomaly signal is a real geophysical signal of a change in landscape surface properties. It shows coherent behavior at regional scales [18], consistent with the regional water storage anomalies observed by GRACE [20]. At finer resolution, the persistence signal is partially explained by loss in forest canopy cover in grid cells that remain ‘forested’ (cover ≥ 60%), but it is not clear to what degree the loss of canopy cover is directly linked with natural forest susceptibility to drought. Analyzing the forest cover loss data [23], we found that the *General Forest* area in the SW quadrant lost about 1000 km^2^ of forest cover per year in 2001–2009, except in the drought year 2005, when it lost about 2000 km^2^ of forest cover. This is consistent with the results of Phillips et al. [13], who analyzed monitoring data across more than 100 plots and found that drought induces a temporary mortality increase in tropical forests, that does not persist longer than about one year. However, these basin-wide forest tree cover loss data are not classified by cause of loss, i.e., human conversion of the forest is not separated from natural tree mortality [23]. In our analysis of the SW quadrant, fewer than 1% of the 1917 *General Forest* grid cells (at 0.2°x0.2°) experienced both a strong 2005 drought and had >+1-sigma 2005–2009 forest cover loss (Table 2), indicating that co-occurrence of drought and forest cover loss was weak at 0.2° resolution. In the SW quadrant, persistent strong reduction in backscatter primarily occurred close to areas of low forest cover (Figs 4A and 6A), and *General Forest* grid cells with forest cover loss >10% during 2005–2009 consistently had a reduction in backscatter (Fig 5B and 5C), despite these not generally being the areas with the most severe drought exposure (Fig 2A). Proximity to low forest cover will often mean proximity to human activity, which may indicate an association with forest degradation [38, 39], and degraded forests may be susceptible to drought impacts under less severe drought conditions than would affect an undisturbed forest. In addition, fast-growing pioneer tree species establishing on formerly cleared or degraded land can be more susceptible to drought due their (typically) low water-use efficiency [40,41].

This new interpretation of the persistent low backscatter is possible because of the new high-resolution pan-Amazon annual forest cover change data set [23], used as a complement to other spatially explicit data sets. Early analyses relied on either a static forest cover [18], or much coarser spatial resolution forest cover change data [22]. Our analysis was unable to identify a clear or single explanation for the persistent low Ku-band microwave backscatter in the southwestern Amazon forest region in the years immediately following the 2005 drought. We assessed multiple variables related to three possible causes for the post-drought persistence in reduced dry-season backscatter–drought, forest cover change, and drier weather conditions post-drought. The multiple lines of evidence assessed here suggest that forest cover loss, possibly a direct or indirect impact of the drought, and lingering drier-than-normal conditions are somewhat more likely contributing factors to low post-drought backscatter than persistent lagged ecophysiological or biogeochemical impacts directly arising from the 2005 drought. We conclude that the regional QScat signal is genuine and indicates a widespread forest canopy change, one that cannot be adequately explained by existing remote sensing data. A better understanding can only come from more extensive ground data and/or analysis of frequent high-resolution imagery before and after droughts.
